# Deformable complex network for refining low-resolution X-ray structures

**DOI:** 10.1107/S139900471501528X

**Published:** 2015-10-27

**Authors:** Chong Zhang, Qinghua Wang, Jianpeng Ma

**Affiliations:** a Applied Physics Program, Rice University, Houston, TX 77005, USA; b Verna and Marrs McLean Department of Biochemistry and Molecular Biology, Baylor College of Medicine, One Baylor Plaza, Houston, TX 77030, USA; c Department of Bioengineering, Rice University, Houston, TX 77005, USA

**Keywords:** deformable complex network, low-resolution X-ray refinement, homology modeling

## Abstract

In macromolecular X-ray crystallography, building more accurate atomic models based on lower resolution experimental diffraction data remains a great challenge. Previous studies have used a deformable elastic network (DEN) model to aid in low-resolution structural refinement. In this study, the development of a new refinement algorithm called the deformable complex network (DCN) is reported that combines a novel angular network-based restraint with the DEN model in the target function. Testing of DCN on a wide range of low-resolution structures demonstrated that it constantly leads to significantly improved structural models as judged by multiple refinement criteria, thus representing a new effective refinement tool for low-resolution structural determination.

## Abstract 2

A new refinement algorithm called the deformable complex network that combines a novel angular network-based restraint with a deformable elastic network model in the target function has been developed to aid in structural refinement in macromolecular X-ray crystallography.

## 1. Introduction   

It is often a challenge to refine the atomic structures of macromolecular assemblies owing to their weak diffraction of X-rays. In order to build better structural models based on limited-resolution experimental data, it is desirable to introduce additional restraints such as the conventional stereochemical potential (Engh & Huber, 1991 ▸). In recent studies, following the development of elastic network models (ENMs; Tirion, 1996 ▸; Hinsen, 1998 ▸; Atilgan *et al.*, 2001 ▸; Stember & Wriggers, 2009 ▸), Schröder and coworkers proposed a deformable elastic network (DEN) method (Schröder *et al.*, 2007 ▸, 2010 ▸) for better structural refinement. The DEN method utilizes ‘reference structures’ from homology models (Qian *et al.*, 2007 ▸; Šali & Blundell, 1993 ▸) and a series of virtual ‘springs’ between randomly selected atom pairs with variable equilibrium lengths to guide the refinement process. In principle, any structure with reasonable quality that bears some similarity to the target model (the one to be refined) could be used as a reference structure. Compared with conventional refinement, the DEN method delivered substantial improvements for a wide range of low-resolution structures. However, the DEN method only incorporated one-dimensional information on distances between atom pairs, and neglected potentially valuable information from higher dimensions as well as the interdependence of pairs owing to the interaction of more than two atoms, thus limiting the performance of refinement.

To address the weakness in the DEN method in refining macromolecular structures, in this work we introduce a deformable complex network (DCN) method that combines DEN with additional information obtained from a deformable angular network (DAN). While DEN defines virtual ‘springs’ between selected atom pairs in the reference model (Schröder *et al.*, 2007 ▸), the DAN defines harmonic angles formed by randomly selected atom triplets. Each atom in a triplet is subject to an angular bending potential. The resultant target function used for refinement includes experimental X-ray diffraction data, the conventional stereochemical potential and the DCN energy that combines DAN and DEN.

DCN is deformable owing to the deformability of both the angular part (DAN) and the distance part (DEN). The direction of deformation at a certain refinement step is determined based on the current configuration of the target structure together with the reference structure. The three parameters γ, μ and *w*_DCN_, where γ and μ control the rate of deformation and *w*_DCN_ is the weight of the DCN restraint, are determined by a three-dimensional grid search with the lowest *R*_free_ factor of the final structure as an indicator of the best choice.

Two sets of tests have been used to evaluate the performance of the DCN method. The first set is the refinement of a high-resolution structure of tobacco PR-5d protein (PDB entry 1aun; Koiwa *et al.*, 1999 ▸) at three lower resolutions using its homology model from the plant antifungal protein osmotin (PDB entry 1pcv; Min *et al.*, 2004 ▸) as the reference structure. The deposited 1aun structure serves as the ‘true answer’ and enables additional assessments of the refined structural models based on multiple criteria besides the *R*_free_ value (Brünger, 1992 ▸), such as the all-atom root-mean-square deviation (r.m.s.d.), the global distance test (GDT) (<1 Å) score (Zemla, 2003 ▸) and the template modeling score (TMscore; Zhang & Skolnick, 2004 ▸). The second set is a broader test of re-refining 16 randomly selected low-resolution structures to demonstrate generality. The results from this set of tests show that by using DCN, which merges information independently fetched from DEN and DAN, we achieved additional improvements over the existing DEN method (Schröder *et al.*, 2010 ▸), with a decrease in *R*_free_ of 0.15–1.95% (0.41–6.75% over conventional refinement). In addition, we obtained constant improvements in terms of mitigated overfitting effects, better Ramachandran statistics and higher-quality electron-density maps.

## 2. Methods   

### 2.1. Summary   

For a macromolecular structure that is to be determined, called the target structure, we first performed a *FASTA* search (Pearson & Lipman, 1988 ▸) for each polypeptide chain with *MODELLER* (Šali & Blundell, 1993 ▸). Templates that shared higher sequence identity with the target structure, were longer in length and had higher resolution would be preferable (Supplementary Table S2). Five homologous structure candidates were built on this chosen template, and that with the lowest discrete optimized protein energy (DOPE) score (Shen & Sali, 2006 ▸) was picked as a reference model for this chain. Reference models of different chains can be generated from different sources and adopt any relative positions and orientations, including overlaps. After all or most chains (for multi-chain systems) in the target structure had their reference models constructed, the position and orientation of each of the reference models was determined by molecular replacement using *Phaser* (McCoy *et al.*, 2007 ▸), and the resultant coordinates were merged into one single coordinate file in PDB format and served as the unique reference structure for the whole molecule. DCN excluded interchain interactions in defining deformable angular and elastic network. The DCN model and the corresponding restraints were automatically generated according to a pre-set criteria for angular network triplets and elastic network pairs. These restraints contributed to the term in the total target function described below.

**Figure 1:**
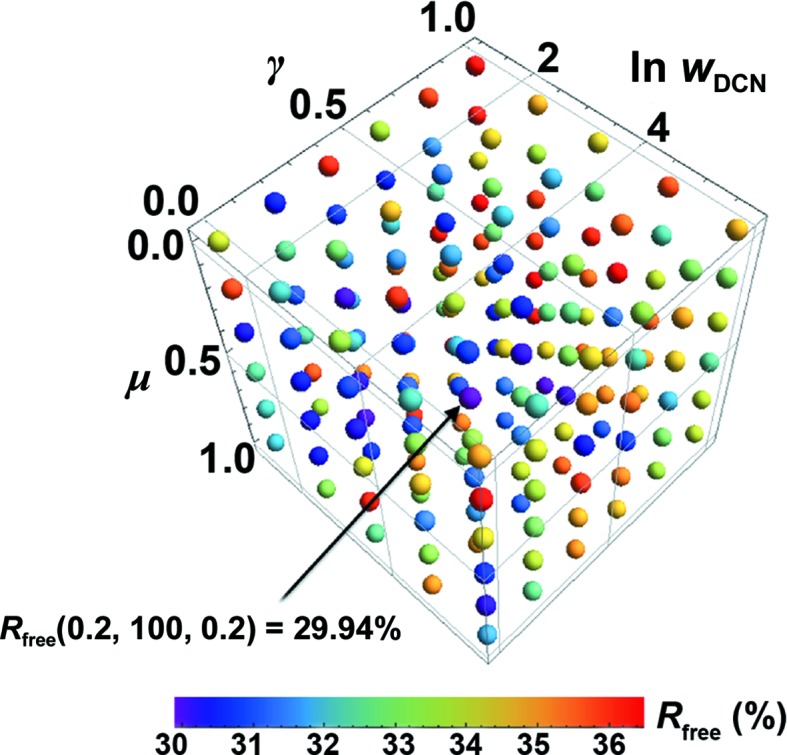
The three-dimensional grid search for optimizing the parameter group (γ, *w*_DCN_, μ). The search is over 180 grid points: (0, 0.2, 0.4, 0.6, 0.8, 1) for γ, (3, 10, 30, 100, 300) for *w*_DCN_ and (0, 0.2, 0.4, 0.6, 0.8, 1) for μ. The particular set that delivers the lowest *R*_free_ for the final structure is the optimum and the corresponding structure is kept for subsequent analysis.

The refinement target function took the form
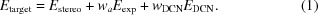
Here, *E*_stereo_ is the usual stereochemical energy. The term *E*_exp_ is the contribution of the experimental diffraction data and *w_a_* is the weight. Typically, the amplitude-based maximum-likelihood function (MLF) was used for *E*_exp_. In the case where experimental phase information was available, the refinement was carried out with the maximum-likelihood Hendrickson–Lattman (MLHL) target function, in which experimental phase contributions were incorporated in the form of Hendrickson–Lattman coefficients (Hendrickson & Lattman, 1970 ▸). *w*_DCN_, the weight of *E*_DCN_, was determined by a specific three-dimensional grid-search method (Fig. 1 ▸). The last term, *E*_DCN_, is the harmonic energy owing to the deviation of selected atom pairs and triplets in the target structure from their corresponding equilibrium values. These values were derived from both the current target structure and the reference structure. Simulated annealing was used as the refinement protocol, with a starting temperature of 3000 K and a cooling rate of 50 K per step. Torsion-angle molecular dynamics were used for dynamics simulation. Refinement with each parameter group was repeated ten times with different random seeds for initial velocity assignments and DCN restraint selections.

### 2.2. Brief description of the DEN model   

The deformable elastic network (DEN; Schröder *et al.*, 2007 ▸, 2010 ▸) is a set of randomly chosen atomic pairs subject to a harmonic potential
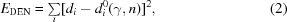
with the summation taken over all atomic pairs in the restraint list. The term *d_i_* is the instantaneous distance for the *i*th pair in the target structure. The equilibrium distance *d_i_* ^0^(γ, *n*) is related to both the reference structure and the target structure. Here, *n* denotes the refinement step and γ is a constant determined in the three-dimensional grid-search procedure (Fig. 1 ▸).

### 2.3. Introduction to the DAN and DCN models   

**Figure 2:**
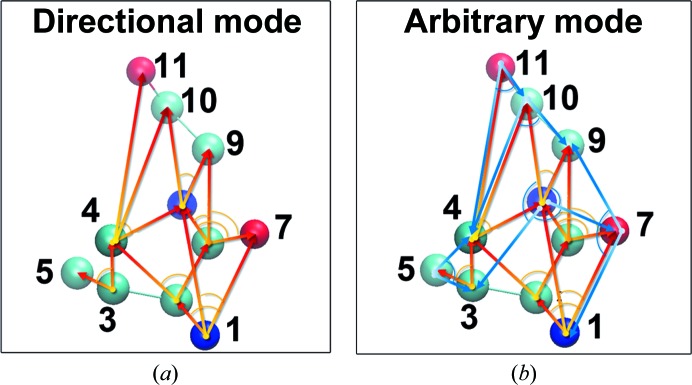
Illustration of the two modes of DAN/DCN. (*a*) The directional mode (D-mode). The value of the atom serial number of the vertex atom is always smaller than that of both tail atoms. For example, for the triplet 3–4–5, in directional mode, the only angle that can be selected is ∠435, where atom 3 is the vertex. Therefore, no more than one angle can be restrained for a given atom triplet and the directional mode tends to ‘spread’ over the entire structure and leads to more diversified atoms being included in the final DAN restraint list. (*b*) The arbitrary mode (A-mode). No restriction is placed for angle selection after an atom triplet is picked. For the triplet 3–4–5, in addition to ∠435 that can be targeted by the directional mode, the arbitrary mode also allows angles such as ∠354, where atom 5 is the vertex, and ∠345, where atom 4 is the vertex. The arbitrary mode includes all possible angles present in a structure. If the cutoff criterion for DAN is flexible enough, two or three angles within the same triplet may be eligible candidates for the selection of final restraints. As a result, in the arbitrary mode it is possible to target angles that have been excluded by directional mode in the first place, but may create less atom diversity. The generated DAN restraint file lists the triplet in the order vertex–first tail–second tail. For both modes, the atom serial number of the first tail is by definition lower than the second to avoid duplication.

In order to define the DCN refinement method, we first introduced a deformable angular network (DAN) model. The DAN model consists of a series of angles, each spanned by two bonds within an atom triplet. The three atoms, of which one is specified as the vertex and the other two as tail atoms, must be present in both the reference and target structures. They also need to satisfy the following additional criteria: (i) all three atoms should be within the same polypeptide chain, (ii) the first and last atom in the triplet should be within a cutoff distance from the middle vertex atom, which is commonly set to be 15 Å, the same as in DEN, (iii) the vertex atom and the tail atom should be no more than ten residues apart and (iv) the vertex angle spanned should be between 60 and 120°. The final angular restraints for refinement were randomly selected from the shortlist, with the number of restraints set to a multiple (one in our study) of the total number of atoms in the target structure. All of these parameters, including the cutoff, residue separation, angle range and restraint-number multiples, were designed to be customizable and to allow finer tuning. We also provided two modes (directional and arbitrary modes) for constructing the restraint list (Fig. 2 ▸).

The harmonic bending energy in DAN is defined as
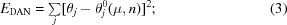
here, the summation was taken over all angle triplets, θ_*j*_ is the instantaneous angle for the *j*th triplet in the target structure and θ_*j*_^0^(μ, *n*) is the corresponding equilibrium angle at a specific (*n*th) refinement step.

DCN was established by combining DEN and DAN. It should be noted that the reference structures of DEN and DAN can be established independently, for example from different homology models. These restraints were considered as unified DCN restraints for use in the total refinement target function.

The DCN potential is the sum of the harmonic stretching energy of DEN and the harmonic bending energy of DAN,
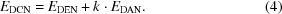
We set the coefficient *k* to 0.01 and the unit of angle was the degree.

We updated **d*_i_* ^0^ and θ_*j*_^0^ every six torsion-angle molecular-dynamics (MD) steps in simulated annealing (when the temperature also drops by 50 K) according to the following equations:
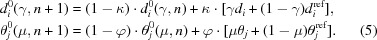
The equilibrium values in the next step for distance and angle, **d*_i_* ^0^(γ, *n* + 1) and θ_*j*_^0^(μ, *n* + 1), were functions of their current equilibrium values, **d*_i_* ^0^(γ, *n*) and θ_*j*_^0^(μ, *n*), their actual instantaneous values, **d*_i_* and θ_*j*_, and the values of the equivalent triplet and pair in the reference model, **d*_i_* ^ref^ and θ_*j*_^ref^. Typically, the initial equilibrium values of the atom pair **d*_i_* ^0^(γ, 0) and the triplet θ_*j*_^0^(μ, 0) were set to these values in the starting structure. The coefficients κ and φ are weights controlling the rate of change between consecutive equilibria. For initial relaxation, κ and φ were set to 0 during the first three macrocycles (refinement protocol). After that, κ and φ were set to a fixed value of 0.1. The values of γ and μ were optimized, together with the weight of the DCN potential *w*_DCN_ (1),[Disp-formula fd1] *via* the three-dimensional grid search (Fig. 1 ▸). The value of *w*_DCN_ was reset to 0 during the last two macrocycles to reduce the bias of the minimum of the target function.

### 2.4. A three-dimensional grid-search scheme for optimizing the parameter set (γ, *w*_DCN_, μ)   

The parameter set (γ, *w*_DCN_, μ) was optimized *via* a three-dimensional grid search through 180 grid points: (0, 0.2, 0.4, 0.6, 0.8, 1) for γ, (3, 10, 30, 100, 300) for *w*_DCN_ and (0, 0.2, 0.4, 0.6, 0.8, 1) for μ (Fig. 1 ▸) At each point, ten refinements with different random seeds were carried out and the result with the lowest *R*_free_ represented the final refined structure at that grid point. The seed controlled the assignment of the initial velocities in dynamics simulation for atoms as well as the selection of DCN restraints from the pair and triplet pool. It should be noted that the final refinement results can depend on the choice of random-number seeds; thus, to ensure consistency, we used the exact integers from 1 to 10 as the ten random seeds throughout this work.

### 2.5. Refinement protocol   

Torsion-angle molecular dynamics (TAMD; Rice & Brünger, 1994 ▸) combined with traditional simulated annealing (Kirkpatrick *et al.*, 1983 ▸) was used as the main refinement protocol (Schröder *et al.*, 2010 ▸). The time step of dynamics simulation was 4 fs. For the annealing process, the initial temperature was set to 3000 K, with a decreasing rate of 50 K per six TAMD steps. Every six TAMD steps can be defined as a ‘microcycle’, which determined the update frequency for both the annealing temperature and the equilibrium values of the DCN restraints. The period in which the temperature decreased from 3000 to 0 K formed a ‘macrocycle’. Each refinement task in this work, including conventional refinement, DEN refinement and DCN refinement, used eight macrocycles. During the first three of them, φ and κ were set to zero rather than 0.1 to allow initial relaxation. The van der Waals radii were decreased to 75% of the original value during several initial macrocycles, together with a reduced van der Waals force constant to facilitate sampling, and were thereafter fully restored in the last two macrocycles. Moreover, the DCN restraint weight was set to zero in the last two macrocycles to reduce the bias in the global minimum of the target function.

Anisotropic overall *B*-factor correction and bulk-solvent correction (Jiang & Brünger, 1994 ▸; Brünger *et al.*, 1998 ▸) were applied to all refinements and no positional minimization was used. For the 16 re-refinement tasks, 50 steps of group *B*-factor minimization with a tenfold increase of the target σ values of the *B*-factor main-chain/side-chain bond/angle restraints were performed, and initial values of *B* factors were reset to 50 Å^2^. Ligands that were not recognized by default by *CNS* (Brünger *et al.*, 1998 ▸; Brunger, 2007 ▸) were explicitly defined as groups for group *B*-factor minimization. For the purposes of appropriate comparison, all refinement parameter settings were kept identical across all test systems. It should be noted that certain parameters, such as the initial annealing temperature, the cooling rate or the multiples of the target σ value for group *B* factors, can also possibly be further optimized for better refinement. Upon the completion of a refinement, all refined structures were sorted according to their values of *R*_free_ and that with the lowest value was chosen for subsequent analysis.

### 2.6. Computation   

Source codes for this approach (*DCN_REF*) were developed under the framework of the *Crystallography and NMR System* (*CNS*; v.1.3; Brunger, 2007 ▸; Brünger *et al.*, 1998 ▸). Computation was carried out on the Shared University Grid at Rice (SUG@R) cluster platform of the Shared Computing Resources (ShareCoRe).

## 3. Results   

### 3.1. Refinement of the tobacco PR-5d protein (PDB entry 1aun) at three lower resolutions   

In this test, we used the crystal structure of the tobacco PR-5d protein (PDB entry 1aun, 1.8 Å resolution; Koiwa *et al.*, 1999 ▸) to allow a systematic assessment of the DCN approach. Its full diffraction data were obtained from the PDB and were then truncated using *CCP*4 (Winn *et al.*, 2011 ▸) to give three lower resolution sets at 3.5, 4.0 and 4.5 Å. These three sets were treated as independent original low-resolution experimental data for subsequent refinement. A homology model (PDB entry 1pcv; 2.3 Å resolution; Min *et al.*, 2004 ▸) was used as the starting structure, the position and orientation of which were determined by molecular replacement using *Phaser* (McCoy *et al.*, 2007 ▸) against each of the three low-resolution data sets. The solutions from the molecular replacement served as the reference structure in the DCN refinement.

To evaluate the performance of the DCN method, we also conducted two other refinements against these three low-resolution data sets. One used the conventional target function combining a stereochemistry potential (Engh & Huber, 1991 ▸) term with the experimental data term (in the form of maximum-likelihood energy; Bricogne & Gilmore, 1990 ▸). The other used the conventional target function in addition to the DEN potential.

**Figure 3:**
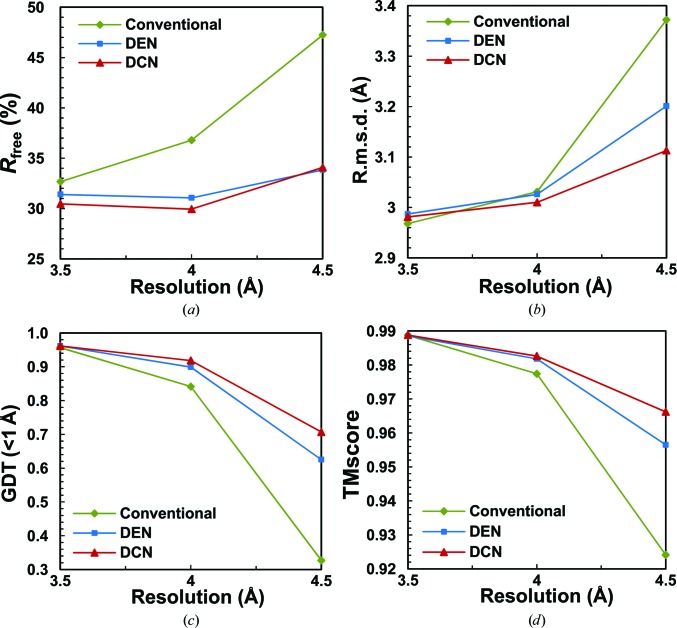
Refinement of the tobacco PR-5d protein at three different lower resolutions. A comparison of *R*_free_ (*a*), r.m.s.d. (*b*), GDT (<1 Å) (*c*) and TMscore (*d*) at three resolutions is shown using conventional refinement (green), the DEN method (blue) and the DCN method (red).

In terms of *R*_free_ values, which measure the agreement between the structural model and X-ray diffraction data (Fig. 3 ▸ *a*), DCN achieved substantial improvements over the DEN method: DCN-refined structural models have a 0.94 and 1.12% lower *R*_free_ than those refined by DEN at 3.5 and 4.0 Å resolution, respectively. At 4.5 Å resolution, the structural models refined by DCN and DEN have similar *R*_free_ values (with the DCN-refined structure having a value 0.24% higher than that of the DEN structure). Compared with the structures refined by the conventional method, the DCN-refined structures are lower in *R*_free_ by 2.21, 6.85 and 13.16% at 3.5, 4.0 and 4.5 Å resolution, respectively (Table 1 ▸, Fig. 3 ▸ *a*).

In addition to the *R*_free_ values, with the 1.8 Å resolution crystal structure 1aun as the ‘true answer’, additional criteria can be used to assess the quality of the refined structure including the all-atom r.m.s.d., the GDT (<1 Å) score (Zemla, 2003 ▸) and the TMscore (Zhang & Skolnick, 2004 ▸). In terms of r.m.s.d. (Fig. 3 ▸ *b*, Table 1 ▸), DCN always outperformed DEN at all three resolutions. For the GDT (<1 Å) score (Fig. 3 ▸ *c*, Table 1 ▸) and the TMscore (Fig. 3 ▸ *d*, Table 1 ▸), DCN consistently delivered the most favorable value among all three refinement approaches. It is important to note that in general the largest improvements provided by DCN are observed at the lowest resolution (4.5 Å); thus, DCN is expected to perform the best for refinement against X-ray data at a resolution limit of 4.0 Å or lower (Table 1 ▸).

### 3.2. Re-refinement of 16 randomly selected low-resolution structures   

We also randomly selected 16 low-resolution all-atom structures (4.0–4.51 Å resolution, 1–14 polypeptide chains, 304–10 941 observed residues; Supplementary Tables S1 and S2) and performed re-refinements. For some structures, the topologies and parameter files of nonstandard ligands, ions and modified residues were obtained from the Hetero-compound Information Center, Uppsala, Sweden (HIC-Up; Kleywegt & Jones, 1998 ▸). To test the performance of DCN, we carried out automatic re-refinements without any manual adjustments. In order to minimize the bias, we reset the DCN potential to zero in the last two of the total of eight refinement macrocycles (see §[Sec sec2]2). As a control, identical protocol and settings were used in DEN and conventional refinements of each of the 16 re-refinements. They generally resulted in better structural models in this work compared with previous work (Supplementary Table S3). These re-refinements serve as the basis for evaluating the performance of our new DCN method.

#### 3.2.1. The *R*_free_ values   

**Figure 4:**
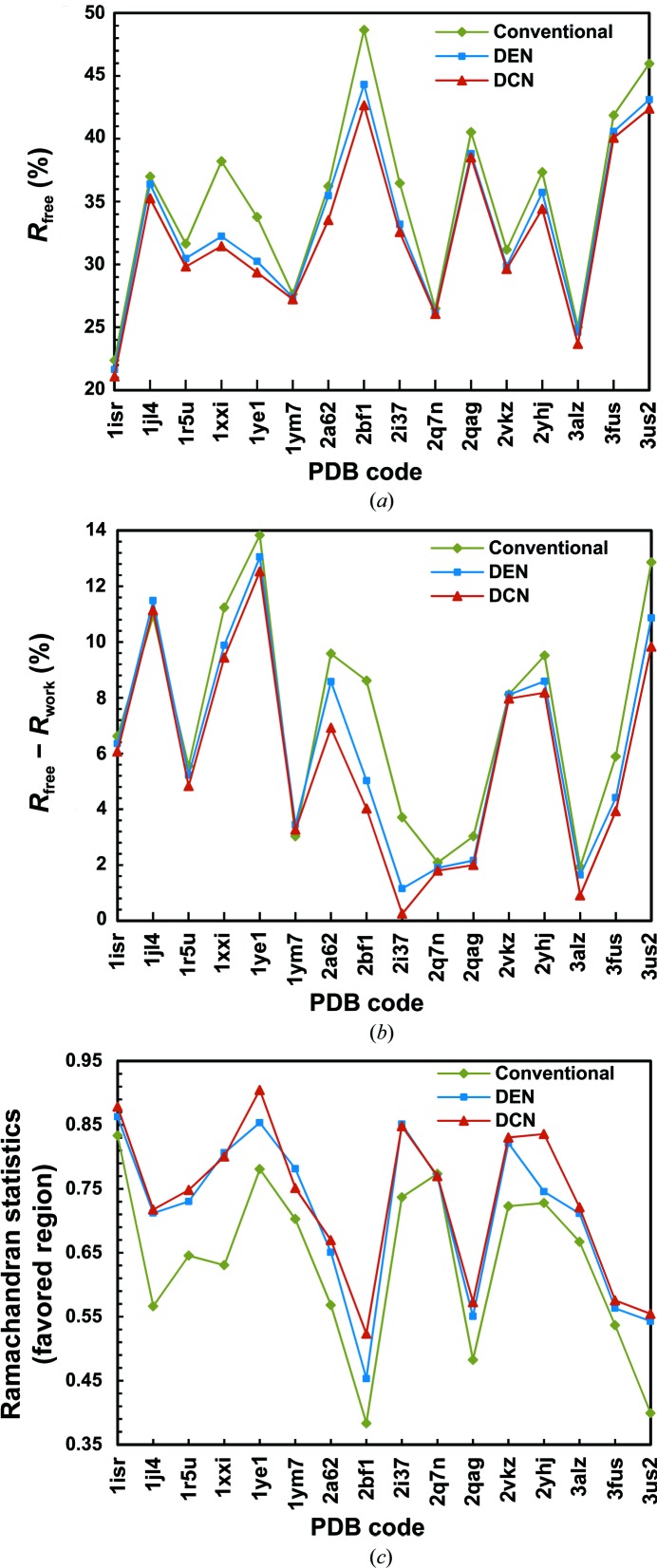
Refinement of 16 randomly selected low-resolution structures. Plots of *R*_free_ (*a*), *R*_free_ − *R*_work_ (*b*) and Ramachandran statistics (*c*) are shown for 16 test systems refined by conventional refinement (green), the DEN method (blue) and the DCN method (red).

*R*_free_ (Brünger, 1992 ▸) was introduced as a cross-validation parameter for the fit between the experimental data and the refined structure, and is ubiquitously used as the primary measure of structure quality in macromolecular crystallography. In our tests of 16 randomly selected low-resolution structures, all of the final *R*_free_ values obtained by DCN were substantially lower than those obtained using the standalone DEN method (ranging between 0.15–1.95%) and more so than those obtained using the conventional method (by 0.41–6.75%) (Table 2 ▸, Fig. 4 ▸ *a*).

#### 3.2.2. Overfitting   

The degree of overfitting can be assessed by the difference between the absolute values of *R*_free_ and *R*_work_. The latter is calculated using the reflections that are involved in the refinement process and is therefore typically smaller than *R*_free_. In most of our test cases (14 of 16), DCN consistently delivered the smallest *R*_free_ − *R*_work_ among all three methods (Table 2 ▸, Fig. 4 ▸). As shown in Table 2 ▸, the case with the most favorable value of *R*_free_ − *R*_work_ for DCN was 0.3% (PDB entry 2i37), whereas for DEN and the conventional method the best cases were 1.2% (PDB entry 2i37) and 1.9% (PDB entry 3alz), respectively. In addition, the averaged *R*_free_ − *R*_work_ from DCN refinement for all 16 test cases is 5.8%, which is 0.6 and 1.5% lower than that from DEN and conventional refinement, respectively (Table 2 ▸).

#### 3.2.3. Ramachandran statistics   

To further evaluate the quality of the refined structures, we carried out structure validation using *MolProbity* (Chen *et al.*, 2010 ▸). Compared with the structures refined by the conventional method, 15 of the 16 DCN-refined structures have a higher percentage of residues that fall in the favored regions of the Ramachandran plot, with a largest increase of 16.9% and an average increase of 9.6% for all 16 cases. Relative to the structures refined by the DEN method, 13 of the 16 DCN-refined structures exhibit a larger percentage of residues in the favored regions, with a largest increase of 9.0% and an average increase of 1.9% (Table 2 ▸, Fig. 4 ▸ *c*). These data collectively suggest greatly enhanced Ramachandran statistics compared with structures refined by the conventional method or the DEN method.

#### 3.2.4. Electron-density maps   

**Figure 5:**
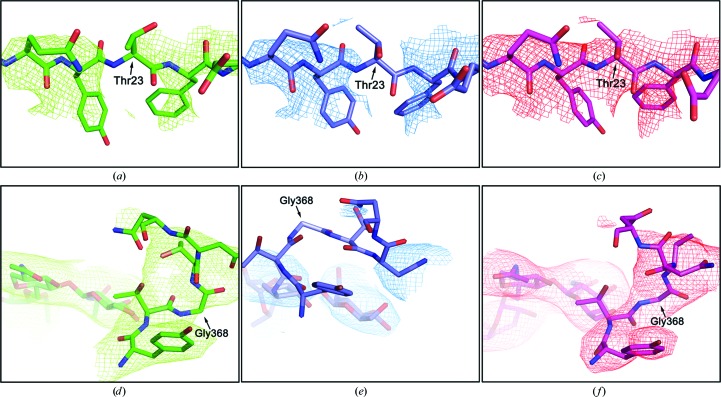
Comparison of structures refined by different methods and their corresponding phase-combined σ-weighted 2*F*_o_ − *F*_c_ electron-density maps. (*a*, *b*, *c*) PDB entry 1jl4, (*d*, *e*, *f*) PDB entry 2bf1. The refined structures (stick models) and the corresponding phase-combined σ-weighted 2*F*_o_ − *F*_c_ electron-density maps (mesh) contoured at 1.5σ are shown for conventional refinement (green), the DEN method (blue) and the DCN method (red).

Re-refinement by the DCN method also resulted in improved electron-density maps (Fig. 5 ▸). The phase-combined σ-weighted 2*F*_o_ − *F*_c_ electron-density maps calculated from the experimental amplitudes and model phases are shown in Fig. 5 ▸ for two examples: PDB entries 1jl4 (Figs. 5 ▸ *a*, 5 ▸ *b* and 5 ▸ *c*) and 2bf1 (Figs. 5 ▸ *d*, 5 ▸ *e* and 5 ▸ *f*). In the example of PDB entry 1jl4, the σ-weighted 2*F*_o_ − *F*_c_ electron-density maps from the structural models refined by the conventional method (Fig. 5 ▸ *a*) or the DEN method (Fig. 5 ▸ *b*) both exhibit broken densities around the main-chain atoms of Thr23. In sharp contrast, the map from the structural model refined by the DCN method has clear density around Thr23 (Fig. 5 ▸ *c*). In the second example, PDB entry 2bf1, DEN refinement resulted in an *R*_free_ value that is 4.35% lower than that from the conventional method and the DEN-refined structure displayed large positional shifts in several places on main-chain atoms relative to the structure refined by the conventional method. However, there are regions where the large structural shifts are not supported by electron-density maps (compare Figs. 5 ▸ *d* and Fig. 5 ▸ *e*). In marked contrast, the DCN-refined structure, with an additional decrease in *R*_free_ (by 1.65%) over the DEN method, showed a much better map-coordinate consistency (Fig. 5 ▸ *f*).

## 4. Discussion   

In macromolecular X-ray crystallography, structural refinement based on lower-resolution experimental diffraction data remains a major challenge where new and efficient refinement algorithms are urgently needed. Previous studies have used a DEN model to aid in low-resolution structural refinement (Schröder *et al.*, 2007 ▸, 2010 ▸). In this study, we developed a new refinement algorithm, DCN, that combines the DEN model with a novel angular network-based DAN restraint that exploits higher-dimension interaction networks among atoms. Test of DCN on a wide range of low-resolution structures demonstrated the power of this new method in delivering significant improvements by multiple measures, thus representing a new effective refinement tool for low-resolution structural determination.

For generality, it was our intention to fix many parameters at their default values without any adjustment in this work. We expect that finer tuning of DCN settings will further enhance the performance and robustness of this method. For instance, restraints can be established only for certain regions of the molecule that have sufficiently reliable reference structures available; several angle criteria for the DCN model can be more elaborately tailored to account for the characteristics of individual macromolecular systems. As an example, choosing DAN sequence separation limits of 5 and 8, respectively, delivered an *R*_free_ of 20.75% for PDB entry 1isr and of 28.97% for PDB entry 1ye1, which are about 0.4% lower than using the default value of 10 as shown in Table 2 ▸. The method can also be extended: in cases where the best homology model found in the database does not possess satisfactory sequence identity or resolution, DAN and DEN information for a single chain can be derived from different homology sources. Also, the deformations of angular network and distance network do not need to be synchronized. A more favorable refinement for a given system may emerge when the two networks deform alternatively or with uneven frequencies. Moreover, DCN can be easily implemented in grid computing servers with an online GUI (O’Donovan *et al.*, 2012 ▸), allowing interested users to use it *via* a web portal with ease.

## Supplementary Material

Three supporting tables.. DOI: 10.1107/S139900471501528X/dz5384sup1.pdf

## Figures and Tables

**Table 1:** Comparison of the three methods in refinement of the tobacco PR-5d protein Refinements of the tobacco PR-5d protein (PDB entry 1aun) based on a homology model of the plant antifungal protein osmotin (PDB entry 1pcv) with a sequence identity of 79.51% and an initial all-atom r.m.s.d. of 3.156 to the ‘true structure’. Within each group, the most favorable results [*i.e.* the lowest *R*_free_ and all-atom r.m.s.d. and the highest GDT (1) and TMscore] are indicated in bold and the least favorable results in italics. Overall, DCN led to ten of the 12 best results and none of the worst results. DEN delivered two of the most favorable results (one of which was shared with DCN) and also two of the least favorable results. Conventional refinement produced ten of the worst results and only one of the best results (lowest r.m.s.d. at 3.5 resolution).

Resolution ()	Refinement approach	*R*_free_ (%)	All-atom r.m.s.d. ()	GDT (1) score	TMscore
3.5	Conventional	*32.67*	**2.968**	*0.9567*	0.9887
	DEN	31.40	*2.987*	**0.9615**	*0.9885*
	DCN	**30.46**	2.981	**0.9615**	**0.9888**
4.0	Conventional	*36.79*	*3.031*	*0.8413*	*0.9774*
	DEN	31.06	3.026	0.8990	0.9818
	DCN	**29.94**	**3.010**	**0.9183**	**0.9826**
4.5	Conventional	*47.24*	*3.372*	*0.3269*	*0.9241*
	DEN	**33.84**	3.201	0.6250	0.9565
	DCN	34.08	**3.113**	**0.7067**	**0.9662**

**Table 2:** Comparison of three methods in re-refining 16 low-resolution structures The results of refining 16 low-resolution structures. *R*_free_ and its improvement, *R*_free_ *R*_work_, as well as Ramachandran statistics, are shown. Statistically, of the total of 16 test systems, DCN outperformed DEN in 16 cases (100%) with respect to *R*_free_, 16 (100%) with respect to *R*_free_ *R*_work_ and 13 (81.25%) with respect to Ramachandran statistics. When compared with conventional refinement, these ratios became 100, 87.5 and 93.75%, respectively. The properties of the structure, the experimental data and the reference model for each test system are listed in Supplementary Tables S1 and S2.

		*R*_free_ (%)			DCN improvement (%)		*R*_free_ *R*_work_ (%)			Ramachandran statistics				
PDB code	Resolution ()	Conventional	DEN	DCN	*R*_free_ over conventional	*R*_free_ over DEN	Conventional	DEN	DCN	Conventional	DEN	DCN	DCN conventional	DCN DEN
1isr	4.00	22.37	21.64	21.10	1.27	0.54	6.6	6.4	6.1	0.833	0.863	0.878	0.045	0.015
1jl4	4.30	37.00	36.39	35.25	1.75	1.14	10.9	11.5	11.1	0.567	0.712	0.718	0.151	0.006
1r5u	4.50	31.65	30.48	29.83	1.82	0.65	5.6	5.2	4.8	0.646	0.730	0.748	0.102	0.018
1xxi	4.10	38.21	32.24	31.46	6.75	0.78	11.2	9.9	9.4	0.631	0.806	0.800	0.169	0.006
1ye1	4.50	33.77	30.24	29.36	4.41	0.88	*13.8*	*13.1*	*12.5*	0.781	0.853	0.905	0.124	0.052
1ym7	4.50	27.64	27.39	27.23	0.41	0.16	3.0	3.4	3.3	0.703	0.781	0.751	0.048	0.030
2a62	4.50	36.22	35.48	33.53	2.69	1.95	9.6	8.6	6.9	0.568	0.651	0.670	0.102	0.019
2bf1	4.00	*48.66*	*44.31*	*42.66*	6.00	1.65	8.6	5.0	4.0	*0.383*	*0.453*	*0.523*	0.140	0.070
2i37	4.15	36.46	33.20	32.57	3.89	0.63	3.7	1.2	0.3	0.737	0.851	0.848	0.111	0.003
2q7n	4.00	26.49	26.21	26.06	0.43	*0.15*	2.1	1.9	1.8	0.774	0.768	0.770	0.004	0.002
2qag	4.00	40.52	38.81	38.52	2.00	0.29	3.0	2.2	2.0	0.483	0.551	0.573	0.090	0.022
2vkz	4.00	31.17	29.88	29.64	1.53	0.24	8.1	8.1	8.0	0.723	0.822	0.830	0.107	0.008
2yhj	4.00	37.34	35.73	34.42	2.92	1.31	9.5	8.6	8.2	0.728	0.746	0.836	0.108	0.090
3alz	4.51	25.01	24.61	23.67	1.34	0.94	1.9	1.6	0.9	0.667	0.712	0.721	0.054	0.009
3fus	4.00	41.87	40.57	40.07	1.80	0.50	5.9	4.4	3.9	0.537	0.563	0.576	0.039	0.013
3us2	4.20	45.97	43.11	42.39	3.58	0.72	12.9	10.9	9.8	0.399	0.543	0.555	0.156	0.012
Average	4.20	35.02	33.14	32.36	2.66	0.78	7.3	6.4	5.8	0.635	0.713	0.731	0.096	0.019
Minimum	4.00	22.37	21.64	21.10	0.41	0.15	1.9	1.2	0.3	0.383	0.453	0.523	0.004	0.030
Maximum	4.51	48.66	44.31	42.66	6.75	1.95	13.8	13.1	12.5	0.833	0.863	0.905	0.169	0.090

## References

[bb1] Atilgan, A. R., Durell, S. R., Jernigan, R. L., Demirel, M. C., Keskin, O. & Bahar, I. (2001). *Biophys. J.* **80**, 505–515. 10.1016/S0006-3495(01)76033-X PMC1301252 11159421

[bb2] Bricogne, G. & Gilmore, C. J. (1990). *Acta Cryst.* A**46**, 284–297.

[bb3] Brünger, A. T. (1992). *Nature (London)*, **355**, 472–475. 10.1038/355472a0 18481394

[bb4] Brunger, A. T. (2007). *Nature Protoc.* **2**, 2728–2733. 10.1038/nprot.2007.406 18007608

[bb5] Brünger, A. T., Adams, P. D., Clore, G. M., DeLano, W. L., Gros, P., Grosse-Kunstleve, R. W., Jiang, J.-S., Kuszewski, J., Nilges, M., Pannu, N. S., Read, R. J., Rice, L. M., Simonson, T. & Warren, G. L. (1998). *Acta Cryst.* D**54**, 905–921. 10.1107/s0907444998003254 9757107

[bb6] Chen, V. B., Arendall, W. B., Headd, J. J., Keedy, D. A., Immormino, R. M., Kapral, G. J., Murray, L. W., Richardson, J. S. & Richardson, D. C. (2010). *Acta Cryst.* D**66**, 12–21. 10.1107/S0907444909042073 PMC2803126 20057044

[bb7] Engh, R. A. & Huber, R. (1991). *Acta Cryst.* A**47**, 392–400.

[bb8] Hendrickson, W. A. & Lattman, E. E. (1970). *Acta Cryst.* B**26**, 136–143.

[bb9] Hinsen, K. (1998). *Proteins*, **33**, 417–429. 10.1002/(sici)1097-0134(19981115)33:3<417::aid-prot10>3.0.co;2-8 9829700

[bb10] Jiang, J.-S. & Brünger, A. T. (1994). *J. Mol. Biol.* **243**, 100–115. 10.1006/jmbi.1994.1633 7932732

[bb11] Kirkpatrick, S., Gelatt, C. D. & Vecchi, M. P. (1983). *Science*, **220**, 671–680. 10.1126/science.220.4598.671 17813860

[bb12] Kleywegt, G. J. & Jones, T. A. (1998). *Acta Cryst.* D**54**, 1119–1131. 10.1107/s0907444998007100 10089488

[bb13] Koiwa, H., Kato, H., Nakatsu, T., Oda, J., Yamada, Y. & Sato, F. (1999). *J. Mol. Biol.* **286**, 1137–1145. 10.1006/jmbi.1998.2540 10047487

[bb14] McCoy, A. J., Grosse-Kunstleve, R. W., Adams, P. D., Winn, M. D., Storoni, L. C. & Read, R. J. (2007). *J. Appl. Cryst.* **40**, 658–674. 10.1107/S0021889807021206 PMC2483472 19461840

[bb15] Min, K., Ha, S. C., Hasegawa, P. M., Bressan, R. A., Yun, D.-J. & Kim, K. K. (2004). *Proteins*, **54**, 170–173. 10.1002/prot.10571 14705035

[bb16] O’Donovan, D. J., Stokes-Rees, I., Nam, Y., Blacklow, S. C., Schröder, G. F., Brunger, A. T. & Sliz, P. (2012). *Acta Cryst.* D**68**, 261–267. 10.1107/S0907444912001163 PMC3282622 22349228

[bb17] Pearson, W. R. & Lipman, D. J. (1988). *Proc. Natl Acad. Sci. USA*, **85**, 2444–2448. 10.1073/pnas.85.8.2444 PMC280013 3162770

[bb18] Qian, B., Raman, S., Das, R., Bradley, P., McCoy, A. J., Read, R. J. & Baker, D. (2007). *Nature (London)*, **450**, 259–264. 10.1038/nature06249 PMC2504711 17934447

[bb19] Rice, L. & Brünger, A. T. (1994). *Proteins*, **19**, 277–290. 10.1002/prot.340190403 7984624

[bb20] Šali, A. & Blundell, T. L. (1993). *J. Mol. Biol.* **234**, 779–815. 10.1006/jmbi.1993.1626 8254673

[bb21] Schröder, G. F., Brunger, A. T. & Levitt, M. (2007). *Structure*, **15**, 1630–1641. 10.1016/j.str.2007.09.021 PMC2213367 18073112

[bb22] Schröder, G. F., Levitt, M. & Brunger, A. T. (2010). *Nature (London)*, **464**, 1218–1222. 10.1038/nature08892 PMC2859093 20376006

[bb23] Shen, M. & Sali, A. (2006). *Protein Sci.* **15**, 2507–2524. 10.1110/ps.062416606 PMC2242414 17075131

[bb24] Stember, J. N. & Wriggers, W. (2009). *J. Chem. Phys.* **131**, 074112. 10.1063/1.3167410 PMC2748695 19708737

[bb25] Tirion, M. M. (1996). *Phys. Rev. Lett.* **77**, 1905–1908. 10.1103/PhysRevLett.77.1905 10063201

[bb26] Winn, M. D. *et al.* (2011). *Acta Cryst.* D**67**, 235–242.

[bb27] Zemla, A. (2003). *Nucleic Acids Res.* **31**, 3370–3374. 10.1093/nar/gkg571 PMC168977 12824330

[bb28] Zhang, Y. & Skolnick, J. (2004). *Proteins*, **57**, 702–710. 10.1002/prot.20264 15476259

