# Supplementary material for: Deformable complex network for refining low-resolution X-ray structures
Source: Acta Crystallogr D Biol Crystallogr. 2015 Oct 27;71(Pt 11):2150–7. doi: 10.1107/S139900471501528X (PMC4631475; doi:10.1107/S139900471501528X)
Supplement: Supplementary file 1 [file d-71-02150-sup1.pdf]

BIOLOGICAL CRYSTALLOGRAPHY

Volume 71 (2015)

Supporting information for article:

Deformable complex network for refining low-resolution X-ray structures

Chong Zhang, Qinghua Wang and Jianpeng Ma

Table S1 Structure properties of 16 randomly selected low-resolution systems for re-refinements

The sequence length varies from 316 to 11,814 and represents a broad range of proteins with various sizes. Deposited values of R_{free} and R_{work} were directly taken from the PDB header.

PDB ID	Resolution (Å)	Number of Chains	Sequence Length	No. of Observed Protein Residues	No. of All Observed Residues*	Ramachandran Statistics of Deposited Structure	Deposited R_{free} (%)	Deposited R_{work} (%)
1ISR	4.00	1	490	448	451	0.948	**25.9**	23.7
1JL4	4.30	4	557	557	557	0.922	*45.3*	*42.0*
1R5U	4.50	11	4259	3517‡	3527	0.805	37.3	34.5
1XXI	4.10	10	3562	3532	3544	0.937	36.9	36.6
1YE1	4.50	4	574	574	772	0.968	34.3	29.5
1YM7	4.50	4	2756	2422	2422	0.899	27.9	**22.4**
2A62	4.50	1	322	319	325	0.749	34.6	27.1
2BF1	4.00	1	316	304	354	*0.680*	38.8	38.5
2I37	4.15	3	1044†	954	975	0.896	38.2	37.7
2Q7N	4.00	4	1336	1320	1365	0.793	28.7	23.7
2QAG	4.00	3	1206	702	705	0.895	39.2	37.6
2VKZ	4.00	6	11814	10941	10947	0.935	26.8	26.8
2YHJ	4.00	2	638	570	570	**0.977**	30.0	24.7
3ALZ	4.51	2	630	526	528	0.812	33.8	32.6
3FUS	4.00	1	316	304	359	0.700	35.4	34.6
3US2	4.20	14	1624	1500	1584	0.886	33.4	32.6
Average	4.20	4.4	1965	1781	1812	0.863	34.2	31.5
Minimum	4.00	1	316	304	325	*0.680*	**25.9**	**22.4**
Maximum	4.51	14	11814	10941	10947	**0.977**	*45.3*	*42.0*

* All observed residues denote the sum of residue entries of protein, nucleic, heterogen, solvent that are observed and used in the refinement.

† Sequence length of 2I37 in PDB website is, however, recorded as 1047. This is because three modified residues of ACE were categorized as heterogen entries in the PDB structure file, but denoted as 'X' and included in the FASTA sequence file. These residues did not take part in the homology modeling process, did not have a corresponding residue in the reference structure, and as a result were not counted into sequence length or protein backbone residues.

‡ Chain M of 1R5U consists of unknown residues (UNK) and was excluded before refinement. Therefore number of protein residues is smaller than sequence length minus number of missing residues.

Table S2 Properties of experiment data and reference structures in re-refinements of 16 randomly selected low-resolution structures

Diffraction data were fetched from PDB and converted into CNS recognized hkl file with no modification. For data set without explicitly given R_{free} flag, a free data set was generated for 5% of the total diffractions. For several test systems (*e.g.* 1JL4), there existed diffraction data with resolution higher than that given in the PDB header. Those hkl data were excluded from the refinement and we only used the portion of data that agreed with the published resolution. Reference structures were chosen according to several preferences stated in the "Methods" section. The sequence identity and resolution values in the table were linearly averaged by chain length according to FASTA sequence, with information of resolution and identity of each chain's corresponding template.

PDB ID	Resolution (Å)	Total No. of Diffractions		No. of Diffractions per Residue		Reference Structure	
		Working	Free	Working	Free	Resolution (Å)	Sequence Identity
1ISR	4.00	6628	552	14.70	1.22	2.20	99.8%
1JL4	4.30	5880	645	10.56	1.16	2.25	94.7%
1R5U	4.50	56023	1721	15.88	0.49	2.28	79.5%
1XXI	4.10	35818	4020	10.11	1.13	2.64	99.8%
1YE1	4.50	3442	354	*4.46*	*0.46*	**1.43**	99.0%
1YM7	4.50	23110	1210	9.54	0.50	2.60	39.4%
2A62	4.50	3929	323	12.09	0.99	2.00	**100.0%**
2BF1	4.00	5842	280	16.50	0.79	1.99	*37.0%*
2I37	4.15	11807	645	12.11	0.66	2.20	**100.0%**
2Q7N	4.00	35237	1852	25.81	1.36	2.06	74.1%
2QAG	4.00	40358	2124	**57.25**	**3.01**	2.60	69.5%
2VKZ	4.00	160231	8547	14.64	0.78	*3.10*	96.0%
2YHJ	4.00	11151	555	19.56	0.97	1.75	**100.0%**
3ALZ	4.51	13413	703	25.40	1.33	2.73	92.2%
3FUS	4.00	5841	279	16.27	0.78	2.20	39.1%
3US2	4.20	14037	744	8.86	0.47	1.82	81.3%
Average	4.20	27047	1535	17.11	1.01	2.24	81.3%
Minimum	4.00	3442	279	*4.46*	*0.46*	**1.43**	*37.0%*
Maximum	4.51	160231	8547	**57.25**	**3.01**	*3.10*	**100.0%**

Table S3 Comparison of some common cases between this work and previous work (Schroder *et al*, 2010).

Results of Conventional and DEN were substantially improved in this work due to the inclusion of ligands during the refinement. For most cases, improvement by DEN over Conventional was also larger. Performance of the DCN approach was evaluated against that of DEN and the conventional method obtained in this study.

PDB ID	Ligands not defined in CNS	Approach	R_{free} (this work, %)	R_{free} (previous work, %)	Improvement (%)
1ISR	GD	Conventional	22.3	23.7	1.4
		DEN	21.6	23.3	1.7
1YE1	HEM	Conventional	33.8	35.0	1.2
		DEN	30.2	31.2	1.0
1XXI	ADP	Conventional	38.2	46.5	8.3
		DEN	32.2	40.7	8.5
2BF1	BMA,NDG	Conventional	48.7	49.2	0.5
		DEN	44.3	47.9	3.6
2QAG	GTP,GDP	Conventional	40.5	40.1	-0.4
		DEN	38.8	39.2	0.4
2VKZ	CER,FMN	Conventional	31.2	33.7	2.5
		DEN	29.9	32.7	2.8
Average			34.3	36.9	2.6
